# Aberration-free aspherical lens shape for shortening the focal distance of an already convergent beam

**DOI:** 10.1107/S1600577517011808

**Published:** 2017-10-06

**Authors:** John P. Sutter, Lucia Alianelli

**Affiliations:** a Diamond Light Source Ltd, Chilton, Didcot, Oxfordshire OX11 0DE, UK

**Keywords:** X-ray, lens, oval, nanofocusing, aberration

## Abstract

The shapes of single lens surfaces capable of focusing divergent and collimated beams without aberration have already been calculated. However, nanofocusing compound refractive lenses (CRLs) require many consecutive lens surfaces. Here a theoretical example of an X-ray nanofocusing CRL with 48 consecutive surfaces is studied. The surfaces on the downstream end of this CRL accept X-rays that are already converging toward a focus, and refract them toward a new focal point that is closer to the surface. This case, so far missing from the literature, is treated here. The ideal surface for aberration-free focusing of a convergent incident beam is found by analytical computation and by ray tracing to be one sheet of a Cartesian oval. An ‘X-ray approximation’ of the Cartesian oval is worked out for the case of small change in index of refraction across the lens surface. The paraxial approximation of this surface is described. These results will assist the development of large-aperture CRLs for nanofocusing.

## Abstract 2

The ideal lens surface for refocusing an already convergent beam is found to be one sheet of a Cartesian oval. This result is applied to the optimal construction of a compound refractive lens for X-ray nanofocusing.

## 1. Introduction   

Compound refractive lenses (CRLs) have been used to focus X-ray beams since Snigirev *et al.* (1996 ▸) demonstrated that the extremely weak refraction of X-rays by a single lens surface could be reinforced by lining up a series of lenses. As the X-ray focal spot size has been brought down below 1 µm, more lenses have been necessary to achieve the very short focal lengths required. Because the absorption of X-rays in the lens material is generally significant, it thus becomes critical to design the CRL with the shortest length possible for the given focal length in order to minimize the thickness of the refractive material through which the X-rays must pass.

In recent years, designs for novel nanofocusing lenses have been proposed. X-ray refractive lenses will deliver ideally focused beam of nanometer size if the following conditions can be satisfied:

(1) The lens material should not introduce unwanted scattering.

(2) The fabrication process should not introduce shape errors or roughness above a certain threshold.

(3) Absorption should be minimized in order to increase the lens effective aperture.

(4) The lens designs should not introduce geometrical aberrations.

In response to the first three of these conditions, planar micro-fabrication methods including electron beam lithography, silicon etch and LIGA have successfully been used to fabricate planar parabolic CRLs, single-element parabolic kinoform lenses (Aristov *et al.*, 2000 ▸) and single-element elliptical kinoform lenses (Evans-Lutterodt *et al.*, 2003 ▸) from silicon. However, the best focus that can be obtained is strongly dependent on material and X-ray energy. A collimating–focusing pair of elliptical silicon kinoform lenses with a focal length of 75 mm has successfully focused 8 keV photons from an undulator source of 45±5 µm full width at half-maximum (FWHM) into a spot of 225 nm FWHM (Alianelli *et al.*, 2011 ▸). On the other hand, although silicon lenses can be fabricated with very high accuracy, they are too absorbing to deliver a focused beam below 100 nm at energy values below 12 keV, unless kinoform lenses with extremely small sidewalls can be manufactured. As a result, diamond has come to be viewed as a useful material for CRLs. Diamond, along with beryllium and boron, is one of the ideal candidates to make X-ray lenses due to good refractive power, low absorption and excellent thermal properties. Planar refractive lenses made from diamond were demonstrated by Nöhammer *et al.* (2003 ▸). Fox *et al.* (2014 ▸) have focused an X-ray beam of 15 keV to a 230 nm spot using a microcrystalline diamond lens, and an X-ray beam of 11 keV to a 210 nm spot using a nanocrystalline diamond lens. Designs of diamond CRLs proposed by Alianelli *et al.* (2016 ▸) would potentially be capable of focusing down to 50 nm beam sizes. Many technical problems remain to be solved in the machining of diamond; however, our current aim is to provide an ideal lens design to be used when the technological issues are overcome. We assume that technology in both reductive and additive techniques will advance in the coming decade and that X-ray refractive lenses with details of several tens of nanometers will be fabricated. This will make X-ray refractive optics more competitive than they are today for the ultra-short focal lengths. When that happens, lens designs that do not introduce aberrations will be crucial.

The aim of this paper is to define a nanofocusing CRL with the largest possible aperture that can be achieved without introducing aberrations to the focus. The determination of the ideal shape of each lens surface becomes more critical as the desired aperture grows. Suzuki (2004 ▸) states that the ideal lens surface for focusing a plane wave is an ellipsoid, although in fact this is true only if the index of refraction increases as the X-rays cross the surface [see Sanchez del Rio & Alianelli (2012 ▸) and references therein, as well as §2.4 of this paper]. The same author also proposes the use of two ellipsoidal lenses for point-to-point focusing, but a nanofocusing X-ray CRL requires a much larger number of lenses because of the small refractive power and the short focal length. Evans-Lutterodt *et al.* (2007 ▸), in their demonstration of the ability of kinoform lenses to exceed the numerical aperture set by the critical angle, used Fermat’s theorem to calculate the ideal shapes of their four lenses, but explicitly described the shape of only the first lens (an ellipse). Sanchez del Rio & Alianelli (2012 ▸) pointed out the general answer, known for centuries, that the ideal shape of a lens surface for focusing a point source to a point image is not a conic section (a curve described by a second-degree polynomial). Rather, it is a Cartesian oval, which is a type of quartic curve (*i.e.* a curve described by a fourth-degree, or quartic, polynomial). An array of sections of Cartesian ovals is therefore one possible solution to the task of designing an X-ray lens for single-digit nanometer focusing. Previous authors in X-ray optics have not calculated analytical solutions (which do exist), but instead relied on numerical calculations of the roots without asking how well conditioned the quartic polynomial is; that is, how stable the roots of the polynomial are against small changes in its coefficients. However, in this paper it will be shown that finite numerical precision can cause errors in the calculation of the Cartesian oval when the change in refractive index across the lens surface becomes very small, as is usually the case with X-rays. Moreover, it has not been made explicit in the literature when it is reasonable to approximate the ideal Cartesian oval with various conic sections (ellipses, hyperbolas or parabolas). As a result, for the sake of rigor, the authors have considered it worthwhile to find the analytical solutions explicitly. This has not been done before in any recent papers. Finally, Alianelli *et al.* (2015 ▸) state that no analytical solution exists for a lens surface that accepts an incident beam converging to a point and that focuses this beam to another point closer to the lens surface. This paper will concentrate on that very case and will show that in fact such a lens surface can be described by a Cartesian oval. The existence of such solutions removes the necessity of using pairs of lens surfaces of which the first slightly focuses the beam and the second collimates the beam again.

**Figure 1:**
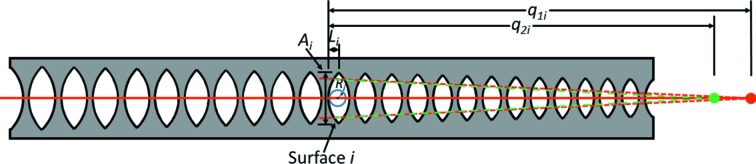
Schematic drawing of an adiabatically focusing CRL for X-rays. The X-ray beam runs along the central axis from left to right. *A*_*i*_, *R*_*i*_ and *L*_*i*_ are, respectively, the geometrical aperture, the radius at the apex and the length along the beam direction of the *i*th lens surface. If the lens surfaces are assumed to be parabolic as in Schroer & Lengeler (2005 ▸), *L*_*i*_ = 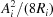. *q*_1*i*_ and *q*_2*i*_ are, respectively, the distance of the object and the distance of the image of the *i*th lens surface from that surface’s apex.

Schroer & Lengeler (2005 ▸) proposed the construction of ‘adiabatically’ focusing CRLs, in which the aperture of each lens follows the width of the X-ray beam as the beam converges to its focus. Fig. 1 ▸ displays a schematic of an adiabatic CRL. A potential example of such a CRL, which would be made from diamond, is given in Table 1 ▸. This example treats X-rays of energy 15 keV, for which diamond has an index of refraction 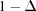 where 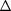 = 3.23 × 10^−6^. The very small difference between the index of refraction of diamond and that of vacuum is typical for X-ray lenses. Calculations of surfaces 2, 24 and 48 of Table 1 ▸ will be demonstrated in the following treatment. The loss of numerical precision when using the exact analytical solutions of the Cartesian oval at small 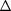 will be avoided by an approximation of the quartic Cartesian oval equation to lowest order in 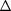. The cubic equation resulting from this ‘X-ray approximation’ will be shown to be numerically stable. The approximate cubic and the exact quartic equation will be shown to agree when the latter is numerically tractable. As in Sanchez del Rio & Alianelli (2012 ▸), conic approximations will be made to the ideal surface in the paraxial case. The results of this paper agree with theirs in showing that Cartesian ovals, even when calculated by the cubic ‘X-ray’ approximation, introduce no detectable aberrations, and that elliptical or hyperbolic lens surfaces introduce less aberration than the usually used parabolic surfaces. However, this paper will also demonstrate that at sufficiently high apertures even the elliptical or hyperbolic approximation will produce visible tails in the focal spot. This is especially true for surface 48, the final surface and the one with the smallest focal length, where the elliptical and hyperbolic approximation produces tails in the focus at an aperture only slightly larger than that given in Table 1 ▸.

At the end of this treatment, the focal spot profiles calculated by ray tracing for the compound refractive lens (CRL) of Table 1 ▸ will be compared with the diffraction broadening that inevitably results from the limited aperture. The absorption in the lens material limits the passage of X-rays through a CRL to an effective aperture *A*_eff_ that is smaller than the geometrical aperture. This in turn restricts the numerical aperture (NA) of a CRL of *N* surfaces to a value *A*_eff_/(2*q*_2*N*_), where *q*_2*N*_ is the distance from the last (*N*th) surface to the final focus. Lengeler *et al.* (1999 ▸) derive a FWHM of 0.75λ/(2NA) for the Airy disk at the focal spot. This yields the diffraction broadening and hence the spatial resolving power of the CRL. Formulas for the effective aperture of a CRL in which all lens surfaces are identical have been derived by Lengeler *et al.* (1998 ▸, 1999 ▸), and Schroer & Lengeler (2005 ▸) have derived formulas for the effective aperture of an adiabatically focusing CRL. Very recently Kohn (2017 ▸) has re-examined the calculation of the effective aperture, surveying the various definitions appearing in the literature and distinguishing carefully between one-dimensionally and two-dimensionally focusing CRLs. In this paper the effective aperture and the numerical aperture will be estimated numerically by ray tracing, taking full account of the absorption to which each ray is subjected along the path from the source to the focus. For simplicity, the lens surfaces will all be assumed to be one-dimensionally focusing parabolic cylinders. It will be shown that the parabola is an adequate approximation to the ideal Cartesian oval within the effective aperture of the CRL in Table 1 ▸.

## 2. Principles   

### 2.1. Definitions and derivation of ideal lens surface   

**Figure 2:**
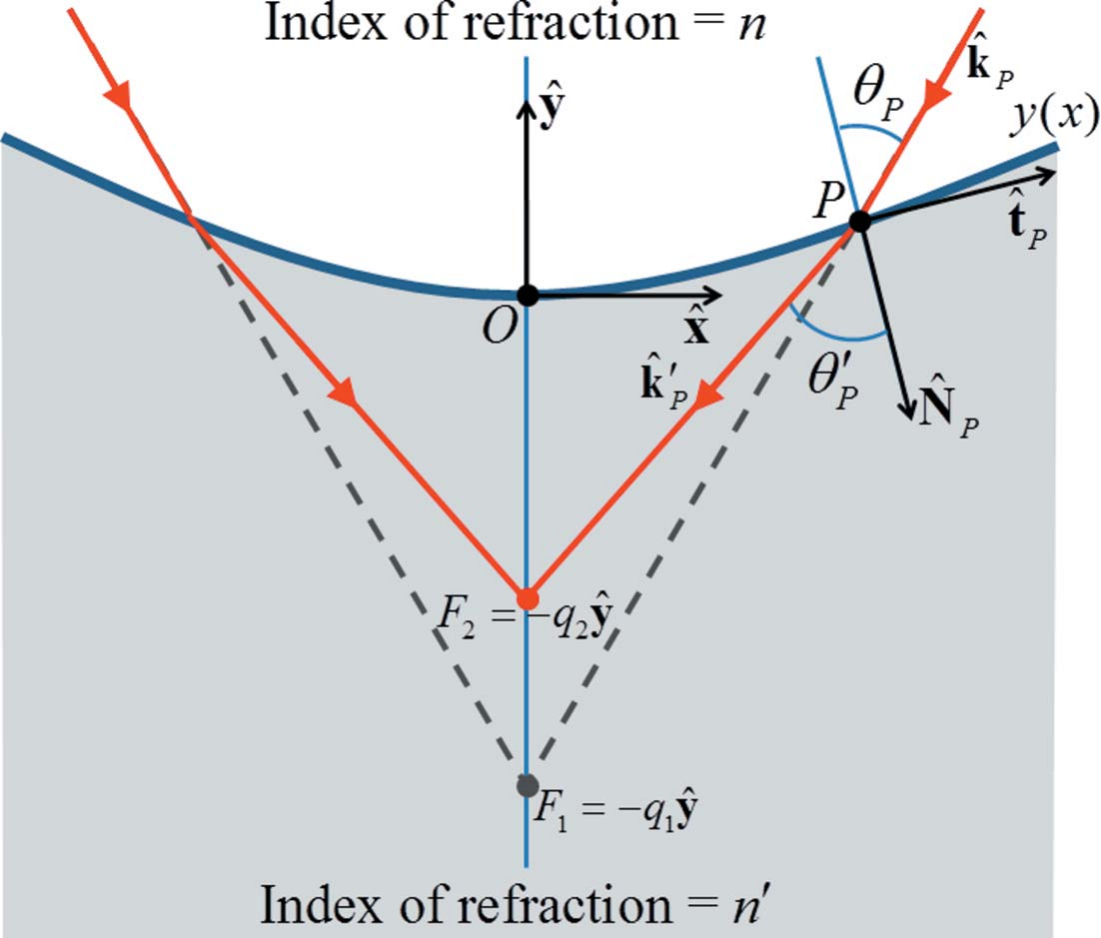
Schematic drawing of surface 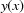 (to be calculated in this paper) across which incident rays in a medium of refractive index *n* converging to a focus 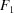 are refracted into rays in a medium of refractive index 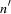 converging to a new closer focus 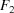. 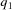 and 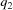 are, respectively, the distances of 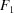 and 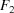 from the coordinate origin *O* along the central axis, which is parallel to 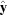. 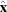 and 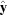 are the coordinate unit vectors. *P* is an arbitrary point along 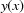. 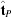 and 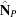 are, respectively, the unit tangent and the unit inward normal to 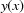 at *P*. 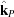 and 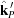 are, respectively, the unit wavevector of the incident ray and the refracted ray passing through *P*. 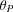 is the angle of the incident ray to the inward normal at *P*, and 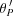 is the angle of the refracted ray to the inward normal at *P*.

The first task of this article is to calculate the exact surface 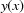 of a lens that bends an already convergent bundle of rays into a new bundle of rays converging toward a closer focus. The required quantities are labelled and defined in Fig. 2 ▸. According to Snell’s Law taken for the rays at an arbitrary point *P*,
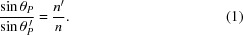
Let the coordinate vector of *P* be 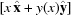. Fig. 2 ▸ shows that
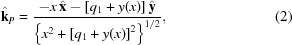


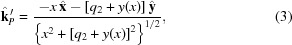


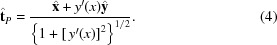
It is also seen in Fig. 2 ▸ that
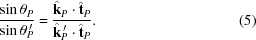
Substitution of equations (1)–(4) into (5) yields the first-order ordinary differential equation
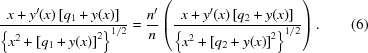
One can rearrange this to find an expression for the surface slope 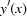, which will be useful for design calculations once a solution for 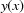 has been obtained,
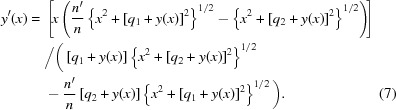
This is a nonlinear differential equation, but nevertheless it can be solved by noticing that the numerators of each fraction of equation (6) are the derivatives of that fraction’s denominator. A simple variable substitution thus presents itself,
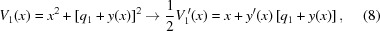


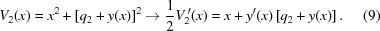
As the initial condition, one may set 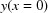 = 0 as shown in Fig. 2 ▸. In that case, 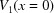 = 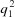 and 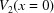 = 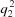. Integration of both sides of equation (6) starting from 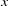 = 0 can then be written
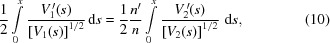
where *s* is a dummy variable. Now, 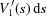 = 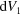 and 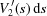 = 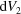, allowing equation (10) to be rewritten in the very simple form
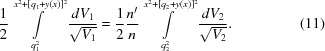
The integrals on both sides of equation (11) are elementary and yield the result
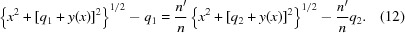
Equation (12) describes a Cartesian oval with the two foci 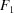 and 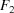 shown in Fig. 2 ▸. Note that the distances of any point on 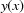 from 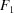 and 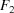 are 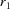 = 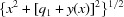 and 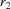 = 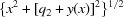, respectively. Equation (12) can then be written in the standard form for a Cartesian oval (Weisstein, 2016 ▸),
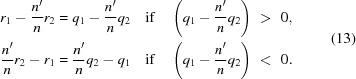
The case 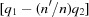 = 0 may be of some interest, and is physically achievable for the problem of this article (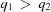) if 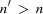. In this case, equation (12) can be squared and re­arranged into
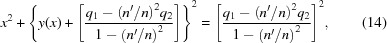
and 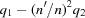 = 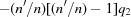 < 0. This is a circle of radius 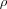 centred at 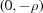, where
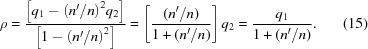



### 2.2. Closed-form solutions of ideal lens surface   

#### 2.2.1. Derivation of algebraic equation   

By adding 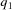 to both sides of equation (12) and then squaring the equation, one obtains
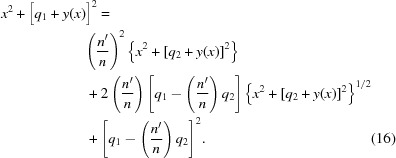
Rearranging this equation to put the radical alone on one side, then squaring it again, yields
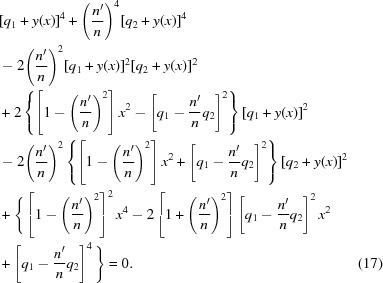
Equation (17) is a quartic polynomial equation in both *x* and *y*. It can be written as a quadratic equation in 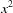, since no odd powers of *x* appear in it, and by using the quadratic formula a closed-form expression of 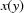 can be calculated. However, the inversion of this function to obtain 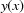 is difficult, and 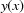 would be far more useful for design calculations of the lens surface. It was thus decided to solve equation (17) for 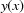 explicitly. Writing equation (17) in powers of *y* yields
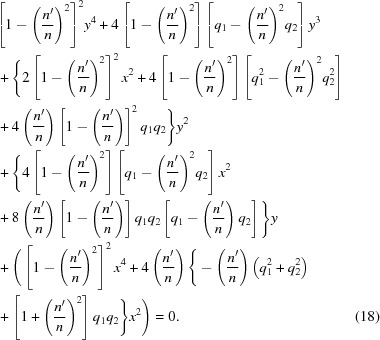
The calculation of 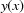 therefore amounts to finding the roots of the quartic polynomial equation (18) for any *x*. As a quartic polynomial equation with real coefficients, equation (18) is guaranteed to have four solutions, of which

– all four may be real, or

– two may be real, while the other two are complex and conjugates of each other, or

– all four may be complex, forming two pairs of complex conjugates.

No more than one of these roots can satisfy the original equation of the Cartesian oval, equation (12). To be physically significant, that root must be real. If equation (18) produces no real root that satisfies equation (12) when calculated at some particular *x*, the ideal lens surface does not exist at that *x*. This raises the possibility that the ideal lens surface may be bounded; that is, it has a maximum achievable aperture.

#### 2.2.2. Calculation of roots of quartic polynomial equation   

Analytical procedures for calculating the roots of cubic and quartic equations were worked out in the 16th century. Nonetheless, explicit solutions of such equations are published so rarely that a detailed description of the method will be useful for the reader. Note that the procedure for quartic equations includes the determination of one root of a cubic equation. Many standard mathematical texts explain the solution of cubic and quartic equations; Weisstein (2016 ▸) has been followed closely here.

The first step in solving a general quartic equation 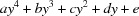 = 0 is the application of a coordinate transformation to a new variable 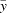 given by
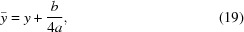
and the division of both sides of the general equation by *a* such that one obtains a ‘depressed quartic’, that is, a quartic with no cubic term, in 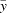. This has the form 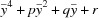 = 0, where
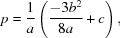


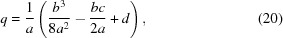


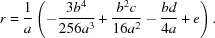
By substituting the coefficients of equation (18) into equations (19) and (20), one obtains the coordinate transformation,
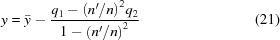
and the depressed equation in 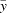, which has the following coefficients,
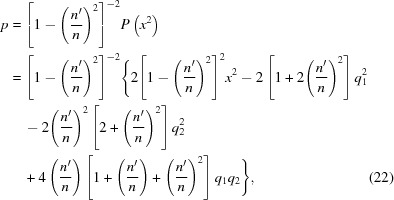


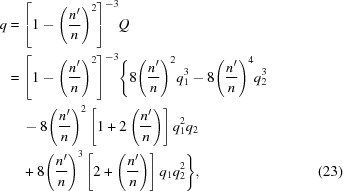


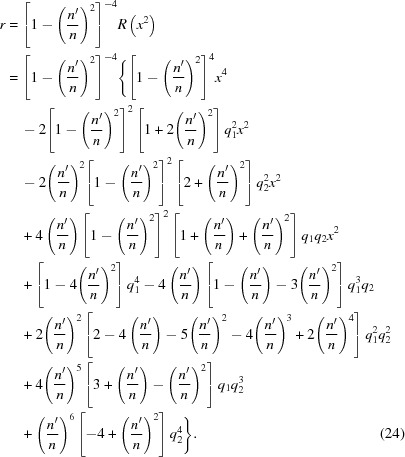
The strategy now is to add to both sides of the depressed equation a quantity 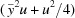, where *u* is a real quantity that will be determined shortly. Knowing that 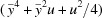 = 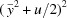, one obtains from the depressed equation the following,
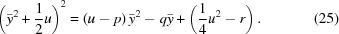
The left side of equation (25) is thus a perfect square. Notice that the right side of equation (25) is a quadratic equation. Therefore it too will be a perfect square if *u* can be chosen to make its two roots equal; that is, if its discriminant *D* equals zero,
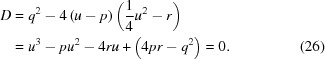
Equation (26) is known as the ‘resolvent cubic’. As a cubic equation with real coefficients, it is guaranteed to have three roots, of which either one or all will be real. The analytical solution of a general cubic equation 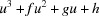 = 0 begins with the calculation of two quantities *A* and *B*, of which the general formula is shown on the left, and the value for the resolvent cubic is shown on the right,
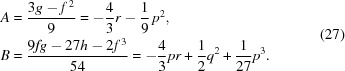
The discriminant of a general cubic equation is 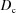 = 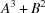. The next step depends on the value of 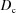.

(i) 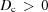. One root of the cubic equation is real and the other two are complex conjugates. The real root is 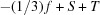, where 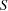 = 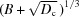 and 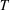 = 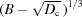. For the resolvent cubic, the real root 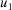 is
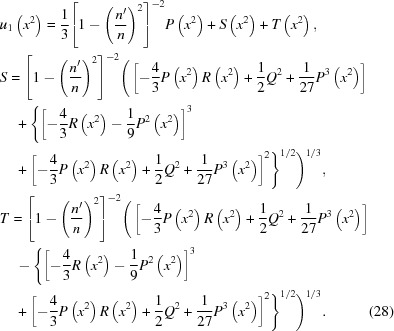



(ii) 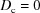. All roots of the cubic equation are real and at least two are equal. *S* and *T* in equation (28) are then equal. Thus, for the resolvent cubic,
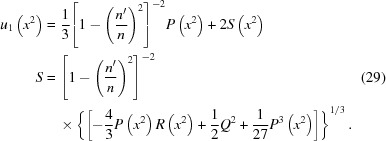



(iii) 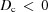. All roots of the cubic equation are real and unequal. In this case, an angle 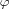 is defined such that
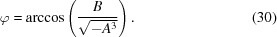
One of the real roots of the general cubic equation is then given by
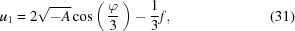
which for the resolvent cubic yields
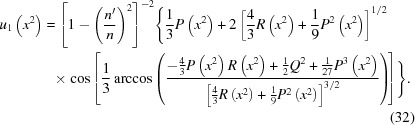
Note that in all these cases one may write 
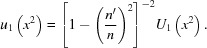
Substitution of 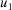 into equation (25) then yields a perfect square on both sides,
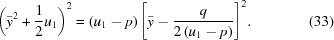
If 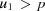, then equation (33) falls into two cases. In the first, one simply equates the square root of both sides,
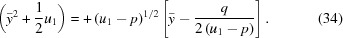
Equation (34) is a quadratic equation in 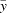. Its two solutions are
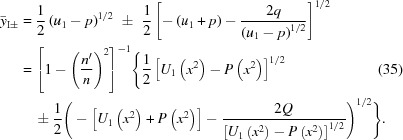
In the second case, one equates the square root of the left side of equation (33) with the negative of the square root of the right side, so that
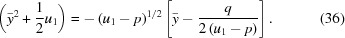
Equation (36), like equation (34), is a quadratic equation in 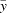. Its two solutions are
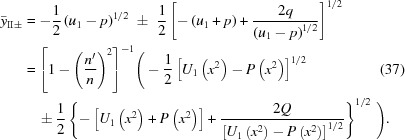


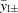
 and 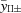 are the four solutions of the depressed quartic. The explicit expressions in *P*, *Q* and 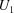 were calculated assuming that 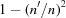 > 0; however, the same expressions are also valid if 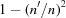 < 0. The only change is that the explicit expression for 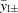 would appear like that for 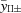 in equation (37), and the explicit equation for 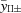 would appear like that for 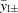 in equation (35). Because this does not affect the solution, it will not be mentioned further.

The solutions of the original quartic equation (18) are easily obtained from equations (35) and (37) by using equation (21),
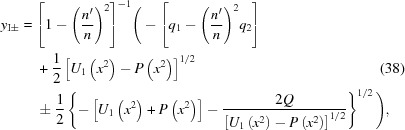


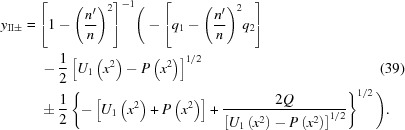
In principle, any of these roots could be the one that satisfies the original equation for the Cartesian oval, equation (12). The simplest way to find this root is to evaluate equations (38) and (39) at 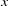 = 0. The root that equals zero there is the correct one. Equation (18) will have one and only one root equal to zero at 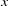 = 0, because then the constant (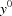) term vanishes but the linear (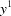) term does not.

If 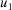 = 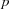, the formulas for the roots become somewhat simpler,
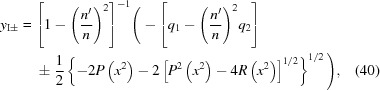


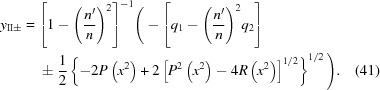
These roots must also be checked to determine which one fulfills equation (12) for the Cartesian oval.

If 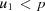, then equation (33) can have a real solution only if both of the squared factors are equal to zero. This would require that 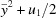 = 0 and 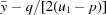 = 0 simultaneously. If that is not possible for the values of *p*, *q* and 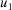 calculated above, then no real solution exists.

**Figure 3:**
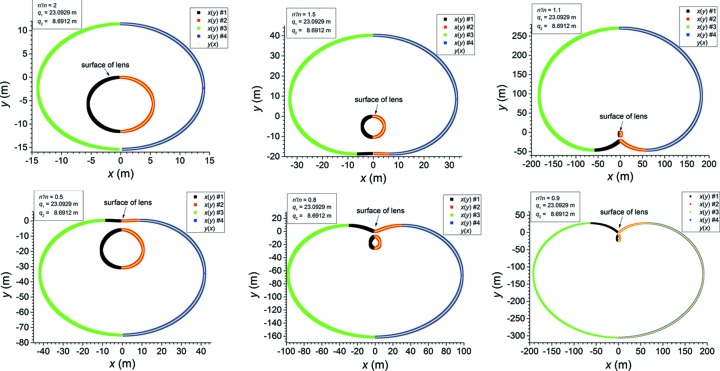
Examples of solutions of the Cartesian oval equation (18) at various values of 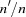 for a representative set of values for 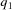 (23.0929 m) and 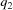 (8.6912 m). The solutions ‘
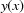
’ were calculated by using the exact equations (38) and (39) for 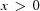; note that 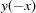 = 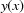. The solutions ‘
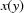
’ were calculated by solving equation (18) as a quadratic equation in 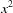 with *y*-dependent coefficients [see equation (45)]. The top row demonstrates three cases in which 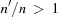 and the bottom row demonstrates three cases in which 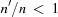. In each row, 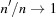 from left to right. The sheet labelled ‘surface of lens’ is the one that fulfills the original lens equation (12).

Fig. 3 ▸ shows a set of examples of the exact solutions in equations (38) and (39) for various values of 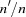. The solutions clearly appear in two disconnected sheets, one inner and one outer. However, only one of these sheets satisfies the original lens equation (12). If 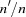 > 1, it is the inner sheet; if 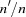 < 1, it is the outer sheet. It is evident that as 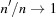 the outer sheet becomes very much larger than the inner sheet. How large the outer sheet becomes at 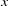 = 0 can be estimated as follows. First, define the following quantities from equations (22)–(24),
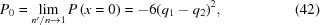


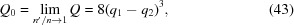


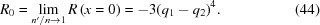
Substitution of these values into equation (27) yields 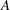 = 0 and 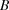 = 0. Thus the discriminant 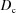 of the resolvent cubic is zero, and from equation (29) one can define 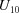 = 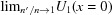 = 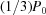. Because 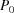 < 0, 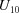 > 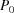 and equations (38) and (39) apply. Letting 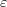 = 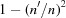 and recalling the initial assumption 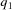 > 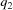, one finds three solutions that remain bounded while the fourth solution 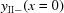 diverges as 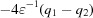. This sensitivity of the fourth root on the exact value of 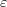 makes the quartic equation (18) ill-conditioned. Therefore the numerical evaluation of equations (38) and (39) is very sensitive to roundoff errors caused by the limited precision in cases in which 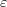 is very small, even though the equations themselves remain theoretically exact. Such cases are not only common but normal in X-ray optics, for which 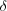 = 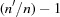 generally has a magnitude on the order of 10^−5^ or less. An approximation that can capture the three bounded roots of equation (18) with high accuracy while ignoring the divergent root is therefore justified.

### 2.3. The ‘X-ray approximation’ to the ideal lens surface   

Equation (18) can be rewritten as a quadratic equation in 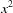 simply by rearranging terms. The quadratic formula can then be applied to determine an equation for 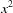 in terms of *y*,
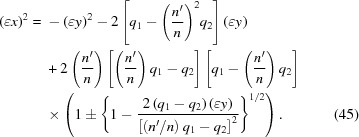
The ‘±’ accounts for the two roots of any quadratic equation. However, if the plus sign is chosen, the resulting equation cannot be satisfied by the condition 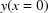 = 0 as is required. Therefore only the minus sign yields a useful set of solutions for the lens surface.

Equation (45) is exact. However, the radical can be expanded by using the binomial theorem if 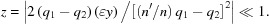
[For the X-ray case where 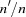 ≃ 1, this condition reduces approximately to 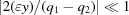.] The binomial theorem yields
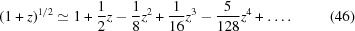
Hence 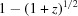 ≃ 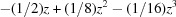 plus higher-order terms that will be discussed later. Note that this has no term in 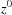. Substituting this into equation (45) and summing terms with the same power of 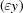 on the right-hand side, one obtains the approximate equation
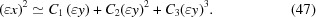
For the linear term on the right-hand side, one obtains
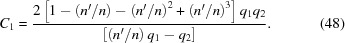
As 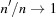, this quantity becomes very small as the terms in the numerator almost cancel out. Equation (48) therefore becomes subject to numerical errors caused by limited precision. However, remembering that in the X-ray case 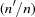 = 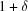 where 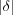 is much less than 1, one can make a power series expansion of 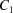 in 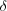. The lowest term of this power series is
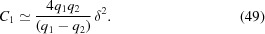
For the quadratic term on the right-hand side of equation (47), one obtains
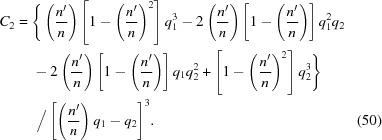
Like 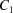, 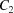 also approaches zero as 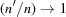. The lowest term of the power series expansion of 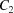 in terms of 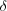 is
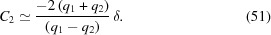
For the cubic term on the right-hand side of equation (47), one obtains
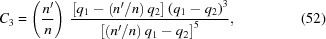
whose power series in terms of 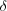 is found simply by setting 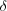 = 0,
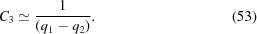
Finally one can calculate the lowest term in the power series expansion of 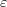, 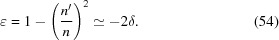
Substituting equations (49), (51), (53) and (54) into equation (47) and keeping only the lowest-order terms in 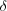 yields
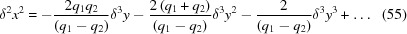
It is justified to keep all terms up to cubic on the right-hand side of equation (55) because all are multiplied by the same power of 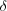. The neglected higher-order terms 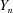 (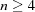) on the right-hand side of equation (55), which arise from the binomial expansion in equation (46), are given to lowest order in 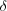 by
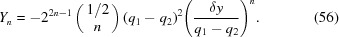
In the X-ray approximation, these terms diminish rapidly with increasing *n*. Therefore the fourth-order term is already much less than the cubic term included in the right-hand side of equation (55), and higher-order terms are smaller still. This justifies the neglect of terms beyond the cubic in equation (55).

In standard form, equation (55) is
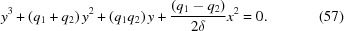
This equation can be solved analytically. When 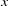 = 0 the roots are trivial: 0, 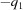 and 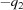. The solutions for general *x* can be determined by the same methods used to calculate the resolvent cubic of the exact equation. The discriminant of equation (57) is 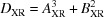, where
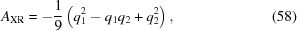


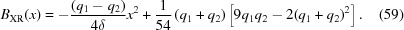
Notice that, since 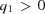 and 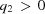, 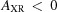 because 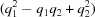 = 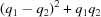 > 0. From these expressions, one obtains
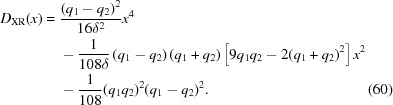
Now one needs to determine the sign of 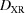 at any given *x*. Notice that 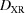 depends quadratically on 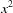. Therefore one can use the quadratic formula to find the values 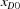 at which 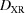 = 0,
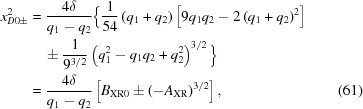
where 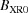 = 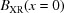. From this one finds that
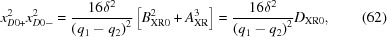
where 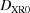 = 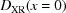. Inspection of equation (60) shows that 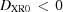 and that therefore 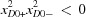, which proves that 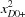 and 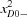 have opposite signs. Equation (61) shows that, if 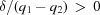, 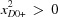 and 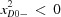, since 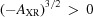. Likewise, if 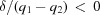, 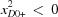 and 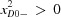. Only the positive squared *x* can yield real values of *x*; the negative squared *x* is discarded. There are thus two values of *x* at which the discriminant 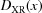 = 0:

(i) 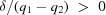.
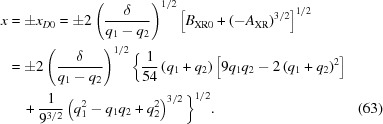



(ii) 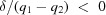.
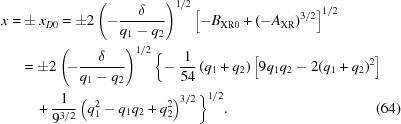
The positive coefficient of the 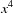 term in equation (60) shows that 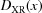 increases with increasing 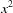. Therefore, the solutions of equation (57) are as follows:

(*a*) 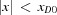, all values of 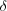. Here the discriminant 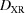 of equation (57) is negative. In this case the three roots of equation (57) are all real and unequal. The calculation of the roots begins with the calculation of an angle 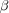 such that
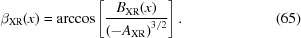
The roots are then
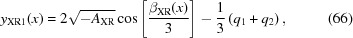


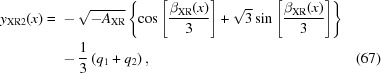


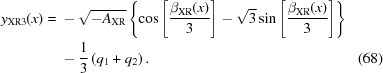
One can now check these roots at 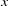 = 0, defining 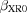 = 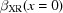. By using the trigonometric identity 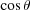 = 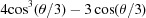, one can show that if 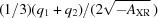 = 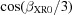, then 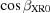 = 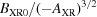, thus satisfying equation (65). One can also use the common trigonometric identity 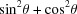 = 1 to find that, for 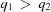 as assumed here, 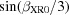 = 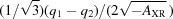. Thus 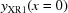 = 0, 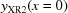 = 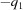 and 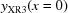 = 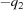, as expected. Note that 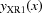 is the solution for the shape of the lens.

(*b*) 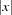 = 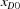, 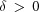 (assuming 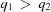). The discriminant 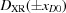 = 0. Therefore the roots are all real and two of them are equal. Substitution of equation (63) into equation (59) shows that in this case 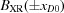 = 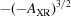. Therefore, according to equation (65), 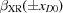 = 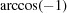 = 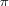. Using equations (66)–(68), one finds the roots
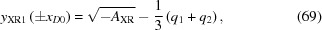


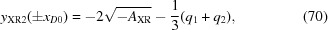


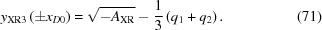
Therefore, in this case, 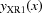 and 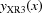 together form the inner sheet of the Cartesian oval. Since we know that 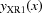 is the desired lens surface, this is consistent with the exact quartic equation.

(*c*) 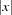 = 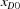, 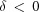 (assuming 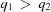). The discriminant 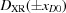 = 0. Therefore the roots are all real and two of them are equal. Substitution of equation (64) into equation (59) shows that in this case 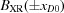 = 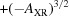. Therefore, according to equation (65), 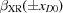 = 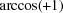 = 0. Using equations (66)–(68), one finds the roots
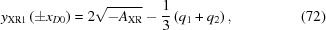


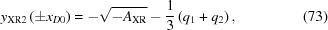


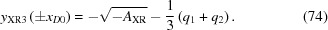
Therefore, in this case, 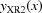 and 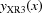 form the inner sheet of the Cartesian oval, and 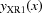 must be on the Cartesian oval’s outer sheet.

(*d*) 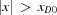, 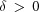 (assuming 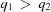). The discriminant 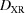 is now positive [see equation (60)]. Therefore only one real root exists. (The other two are complex and hence not physically significant.) This root must join up with 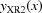 in equation (70). The real root is given by the expression
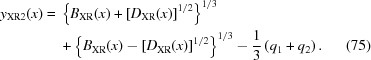
As 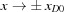, 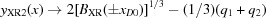 = 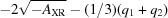, which does indeed join up with equation (70) as expected. Note that this does *not* form part of the solution to the lens surface, but it is included here for completeness.

(*e*) 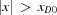, 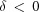 (assuming 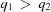). Again, as the discriminant 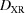 is positive, only one real root exists. This root must join up with 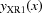 in equation (72),
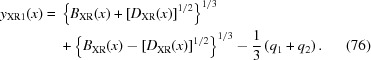
As 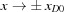, 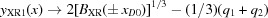 = 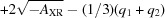, which does indeed join up with equation (72) as expected. This *does* form part of the solution to the lens surface.

**Figure 4:**
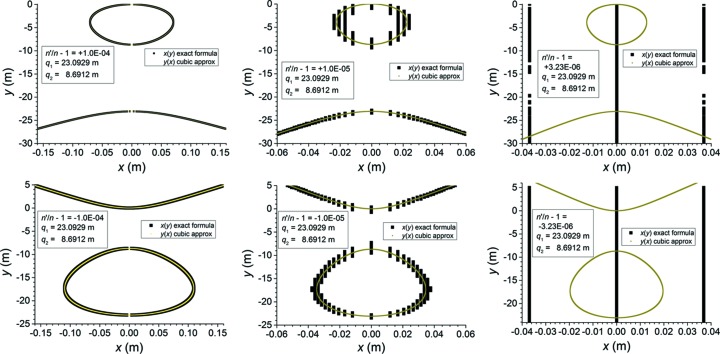
Examples of solutions of the cubic X-ray approximation [equation (57)] at various values of 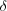 for a representative set of values for 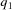 (23.0929 m) and 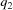 (8.6912 m). The solutions of the cubic approximation are labelled 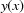. The solutions ‘
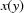
’ were calculated by solving equation (18) as a quadratic equation in 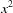 with *y*-dependent coefficients [see equation (45)]. The top row demonstrates three cases in which 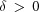 and the bottom row demonstrates three cases in which 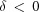. In each row, 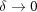 from left to right. Note the loss of numerical precision in the solutions of the exact formula as 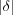 decreases, even while the cubic approximation remains numerically stable.

Examples of the cubic X-ray approximation of the Cartesian oval are shown in Fig. 4 ▸. The outputs displayed in these graphs were calculated using *MATLAB* (MathWorks, 2004 ▸) in the default double precision. At 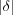 = 10^−4^, the exact equation for the Cartesian oval is still well conditioned enough to deliver stable output, and the X-ray approximation already agrees well with it. It is at values of 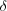 below this that the usefulness of the X-ray approximation becomes obvious. Attempts to use the exact formula result in inconsistent output, while the output of the X-ray approximation remains stable.

**Figure 5:**
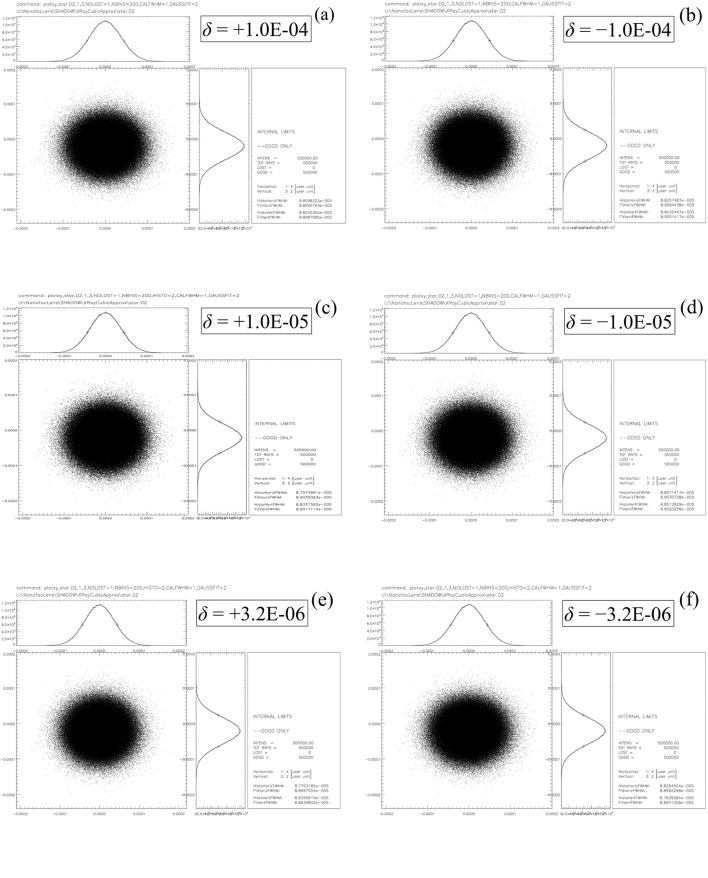
Six ray-tracing simulations performed by *SHADOW3* on the cases displayed in Fig. 4 ▸, except for a slight modification in (*e*) and (*f*) to accommodate the limited precision given by *SHADOW3* to the index of refraction. The values of 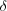 = 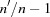 are shown on each diagram. In all simulations, 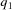 is 23.0929 m and 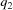 is 8.6912 m as in Fig. 4 ▸. All of the rays originate from a Gaussian source of 1 µm r.m.s. height and width. They are caused by an ideal primary focusing element to converge to the initial focus 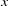 = 0, 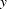 = 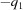. The refractive surface given by the X-ray approximation in each case is calculated on a 501 × 501 mesh of points over an aperture 0.07425 mm × 0.07425 mm. The *SHADOW3* utility *PRESURFACE* is used to determine a spline function over this mesh. The resulting spline is then added to a refractive plane surface, which thus causes the rays from the primary focusing element to converge onto the final focal point 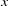 = 0, 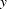 = 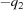. Units of distance are centimetres. See text for further details.

**Figure 6:**
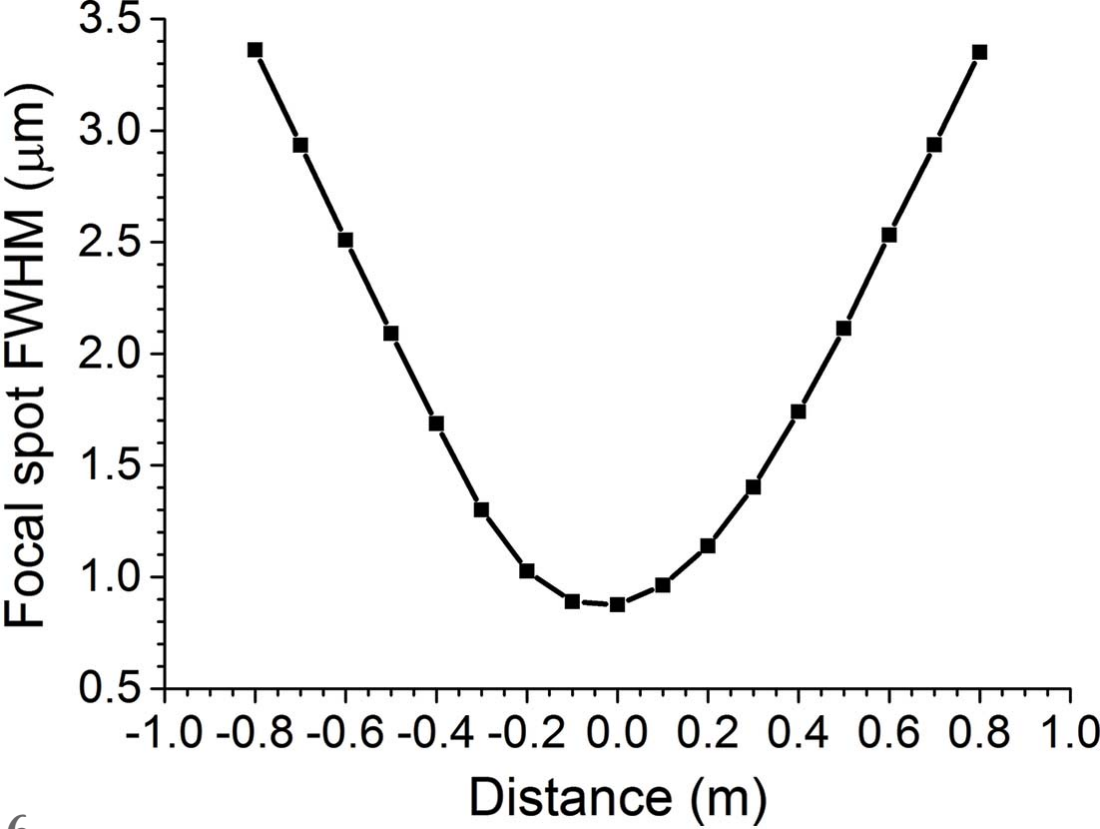
Depth of focus calculation of lens surface calculated by the X-ray approximation. A distance of zero denotes the image point at 8.6912 m downstream from the lens surface.

Fig. 5 ▸ displays a series of *SHADOW3* ray-tracing simulations (Sanchez del Rio *et al.*, 2011 ▸) of the lens surfaces determined by using the X-ray approximation for the six cases in Fig. 4 ▸. All of the 500000 rays originate from a two-dimensional Gaussian source of 1 µm height and width. This is much smaller than a normal synchrotron electron beam source, but was chosen to keep down aberrations that appear when the source size becomes comparable with the lens aperture. The rays are randomly sampled in angle over a uniform distribution of horizontal width 1.6 µrad and vertical width 1.6 µrad. These widths were chosen in order to just exactly cover the full aperture of the lens surface. Two optical elements are used in each simulation. The first optical element is used solely to turn the divergent rays from the source point into a convergent beam. It is a perfectly reflecting, ideally shaped ellipsoidal mirror located 23.10287 m from the source point. This mirror is set to a central grazing incidence angle of 3 mrad. It is shaped so that its source point coincides with the original source of the rays and its image point lies 23.10287 m downstream. The second optical element is the lens surface. It is located 0.010 m downstream from the first optical element. The image point lies 8.6912 m downstream. The basic shape is a plane surface of aperture 0.07425 mm horizontal × 0.07425 mm vertical. To this plane is added a spline interpolation generated by the *SHADOW3* utility *PRESURFACE* from a 501 × 501 mesh of points calculated by *MATLAB* from the X-ray approximation of this section. The index of refraction is taken as constant in the medium upstream from the lens surface and in the medium downstream from the lens surface. Absorption is neglected in both media. The displayed plots are all taken at the final focal point. In all six simulations, the distribution of rays in the image fits well to Gaussians of FWHM very close to 0.886 µm, the geometrical demagnified source size, in both height and width. A calculation of the spot size using the *SHADOW3* utility *RAY_PROP* on 17 frames over a range within ±0.8 m from the image point at 8.6912 m showed that this point was indeed, as required, the point at which the rays converged (see Fig. 6 ▸). It is therefore demonstrated that the X-ray approximation can indeed generate lens surfaces that focus convergent beam.

### 2.4. The paraxial approximation to a conic section   

If the incident rays deviate from the central line 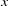 = 0 by only a small amount, the calculations of the ideal lens surface and of the lens surface in the X-ray approximation both show that the value 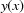 of the lens surface will also be small. In this case, one can assume that the cubic term in equation (47) is much smaller than the quadratic term, thus leading to the condition
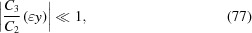
for which the cubic term in equation (47) can be neglected. [Recall that 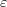 = 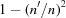 and that 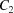 and 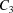 are defined in equations (50) and (52), respectively.] In the X-ray approximation, equation (77) reduces to the simple condition
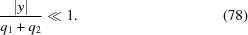
If equation (77) (for the general case) or equation (78) (for the X-ray approximation) is fulfilled, then the paraxial approximation is valid. Equation (47) then reduces to
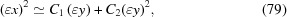
and equation (57) for the X-ray approximation reduces to
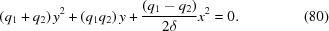
The solutions 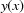 of these equations are conic sections. The type of conic section depends on the sign of the quadratic term 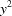. Beginning with general values of 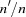, one can complete the square of equation (79) for two cases:

(i) 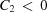. The paraxial approximation to the ideal lens surface is the ellipse
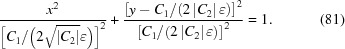



(ii) 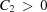. The paraxial approximation to the ideal lens surface is the hyperbola
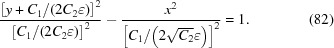



In the X-ray approximation, one can complete the square of equation (80) for two cases:

(i) 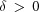. The paraxial approximation to the lens surface is the ellipse
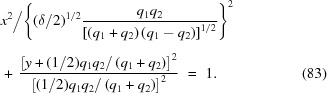



(ii) 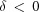. The paraxial approximation to the lens surface is the hyperbola
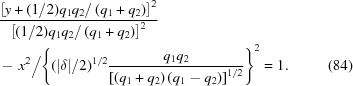
In the limit 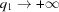 and 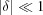, equations (83) and (84) approach the conic sections calculated by Sanchez del Rio & Alianelli (2012 ▸).

An even stricter paraxial approximation is obtained if, in addition to the condition given in equations (77) or (78), one demands that the quadratic 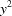 term in equations (79) or (80) be much smaller than the linear *y* term. For general 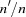, this imposes the additional requirement
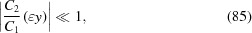
which in the X-ray approximation becomes
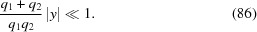
[One can see that equation (86) is in fact more stringent than equation (78) by showing that 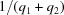 < 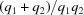 for positive 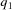 and 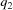.] If the conditions of equations (85) or (86) are fulfilled, the lens surface may be approximated as a parabola. For general 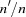, the lens surface is then approximately
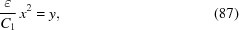
and in the X-ray approximation the lens surface is approximately
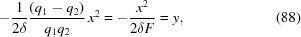
where *F* is the geometrical focal length. Double differentiation of equation (88) yields the well known relationship between the radius *R* and the focal length *F* of a single lens surface in the X-ray approximation, 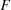 = 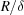.

## 3. Testing the paraxial approximation   

**Figure 7:**
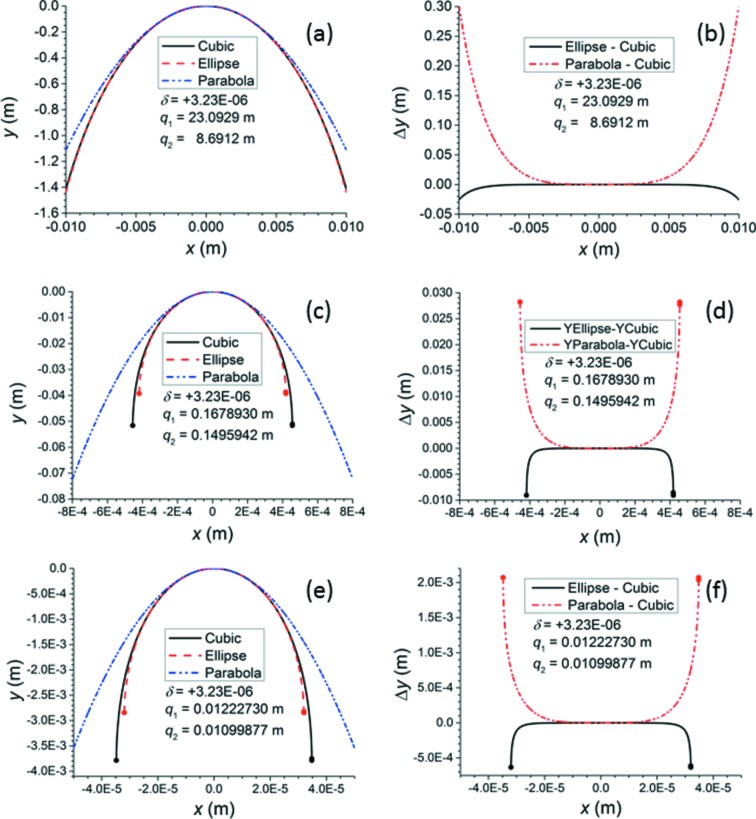
In all plots, 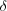 = 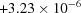. The label ‘Cubic’ means that the X-ray approximation of the ideal Cartesian oval was used to calculate the curve. Refer to Table 1 ▸ for the list of surfaces. (*a*, *c*, *e*) Comparison of cubic curve to paraxial ellipse and parabola of surfaces 2, 24 and 48, respectively. (*b*, *d*, *f*) Deviation of paraxial ellipse and parabola from cubic curve of surfaces 2, 24 and 48, respectively. Solid circles at the ends of a curve indicate that the curve terminates there because the slope 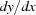 diverges.

**Figure 9:**
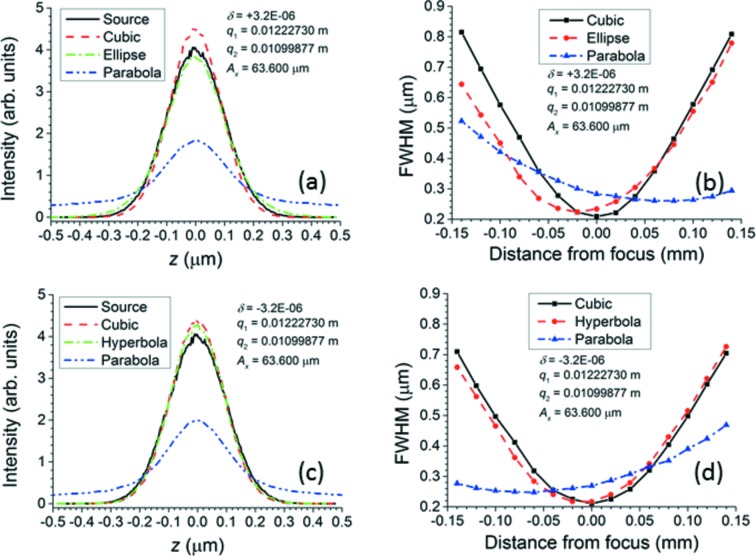
*SHADOW* ray-tracing calculations using surface 48 of Table 1 ▸ over a geometrical aperture *A*_*x*_ of 63.600 µm. The source is a Gaussian with a root mean square width of 0.1 µm. (*a*, *c*) Profiles of source and of focal spots produced by the cubic curve, the paraxial conic section (ellipse/hyperbola) and the parabola for 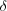 = 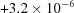 and 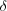 = 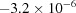, respectively. (*b*, *d*) FWHM of focal spots produced by the cubic curve, the paraxial conic section (ellipse/hyperbola) and the parabola for 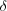 = 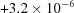 and 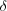 = 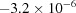, respectively, as a function of distance along the beam from the nominal focus. See text for details.

Figs. 7 ▸ and 8 ▸ demonstrate how, in the paraxial approximation, the best conic section (ellipse for 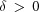, hyperbola for 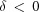) and the best parabola deviate from the X-ray approximation to the ideal Cartesian oval for 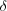 = 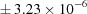, the value for diamond at 15 keV. Surfaces 2, 24 and 48 were selected from Table 1 ▸ to demonstrate that the conic section approximations fail at decreasing apertures as the curvature of the surface increases. As mentioned by previous authors, the parabola deviates from the X-ray approximation at much smaller apertures than does the best ellipse or hyperbola. Each plot’s horizontal axis is scaled to make visible the aperture at which even the best ellipse or hyperbola begins to deviate from the X-ray approximation. Thus, for surface 2, which has an aperture of 74.25 µm, one would expect the parabolic approximation to be sufficient because it matches the X-ray approximation well out to 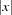 < 2500 µm. For surface 24, which has an aperture of 59.52 µm, the parabolic approximation could still be sufficient, but, as the parabola only matches the X-ray approximation out to 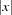 < 200 µm, one might prefer to give this surface an elliptical or hyperbolic shape. For surface 48, which has an aperture of 46.760 µm, even the elliptical/hyperbolic approximation begins to fail at the edges; therefore, this surface must follow the ideal curve. As a result, surface 48 was chosen for the *SHADOW* ray traces of Fig. 9 ▸. To emphasize the improvement offered by the X-ray approximation over the ellipse/hyperbola, the aperture of surface 48 was slightly widened to 63.600 µm, at which Figs. 7 ▸ and 8 ▸ show that the ellipse or hyperbola fails severely at the edges. A value 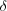 = 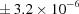 was chosen because of the limited precision given to the index of refraction input in *SHADOW*. In each simulation, 500000 rays were randomly selected from a Gaussian source of root mean square width 0.1 µm (FWHM 0.23548 µm) and uniform angular distribution. Although the chosen size of the source is much smaller than the electron beam sizes of real synchrotron storage rings, it is applied here to approximate a true point source, eliminating aberrations that would appear in the focal spot if the source size were comparable with the lens surface’s aperture. Two optical elements were created. The first was a purely theoretical spherical mirror designed to reflect all rays from the source at normal incidence. This element exists only to produce the necessary convergent beam for the second element, which is the lens surface itself. The second element is situated 12.227 mm upstream from the focus of the spherical mirror. It is simulated with a plane figure to which a spline file generated by the *SHADOW* utility *PRESURFACE* is added. *MATLAB* was used to calculate a cylinder for one-dimensional focusing with 501 points over a width of 46.760 µm in the non-focusing direction and 681 points over a width of 63.600 µm in the focusing direction. The rays in the calculated profiles were sorted into 250 bins according to their position. Figs. 9(*a*) and 9(*c*) ▸ show the beam profiles generated at the nominal focus 10.999 mm downstream from surface 48, comparing them with the original source. The profiles generated by the ellipse or hyperbola are slightly but noticeably lower at the peak and have slightly larger tails than those generated by the X-ray approximation to the ideal curve. The profiles generated by the parabola show a loss of about 50% of the peak intensity and correspondingly severe tails. Depth of focus plots showing the variation of the beam size *versus* the distance along the beam direction from the nominal focus were generated by the *SHADOW3* utility *RAY_PROP* and are displayed in Figs. 9(*b*) and 9(*d*) ▸. The X-ray approximation yields a lens surface that minimizes the beam width at the nominal focus, as required. The FWHM of the beam profile at this minimum is 0.210 µm, which matches the geometrically demagnified source size. The ellipse/hyperbola shifts the minimum of the beam width closer to the lens surface by 10–20 µm, and this minimum is still not quite as small as that achieved by the X-ray approximation. The parabola shifts the minimum of the beam width by about 60–70 µm from the nominal focus, and this minimum is considerably larger than that achieved by either the X-ray approximation or the ellipse/hyperbola. These results again demonstrate that the X-ray approximation can generate lens surfaces that focus convergent beam better than the approximate conic sections can do.

## 4. Diffraction broadening   

**Figure 10:**
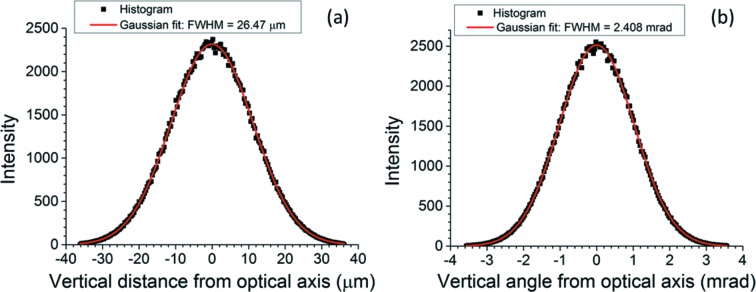
(*a*) Vertical intensity distribution calculated by *SHADOW* ray trace on final lens surface (number 48) of the CRL in Table 1 ▸. (*b*) Vertical angular distribution of intensity calculated by *SHADOW* ray trace at the final focus of the CRL of Table 1 ▸. Each histogram sorts the 500000 rays of the simulation into 200 bins. See text for details.

*SHADOW* was used to calculate the effective aperture of the CRL in Table 1 ▸. 500000 rays of 15 keV energy were created from a point source. They were uniformly distributed in angle so that the entire geometrical aperture of the first lens surface (0.075 mm × 0.075 mm) was illuminated. Each lens surface was assumed to be a parabolic cylinder, focusing in the vertical direction only. All of the lens surfaces except the first were taken to be unbounded so that their limited geometrical apertures would not cut off any of the rays propagating inside the CRL. The lens material is diamond, which for 15 keV X-rays has an index of refraction differing from 1 in its real part by −3.23 × 10^−6^. The linear absorption coefficient μ is 0.282629 mm^−1^. The calculated intensity distribution on the last surface (number 48) and the calculated angular distribution of the intensity converging onto the final focus are displayed in Figs. 10(*a*) and 10(*b*) ▸, respectively. The effective aperture is the FWHM of the plot in Fig. 10(*a*) ▸, 26.47 µm. The corresponding numerical aperture is half the FWHM of the angular plot in Fig. 10(*b*) ▸, 1.204 mrad. The diffraction broadening therefore amounts to 0.75λ/(2NA) = 25.74 nm, which is much less than the focal spot widths in Fig. 9 ▸. Moreover, within the FWHM effective aperture in Fig. 10(*a*) ▸, a parabola is still a sufficiently good approximation to the ideal shape of the final lens surface, as shown in Figs. 7(*e*) and 7(*f*) ▸.

## 5. Conclusions   

The immediate goal of this paper was to prove that an analytical solution, namely a Cartesian oval, exists for a lens surface that is to refocus an incident beam converging to a point into a new beam converging to a point closer to the lens surface. This result serves the long-term goal of designing aberration-free aspherical CRLs that will in future produce X-ray beam spots of 50 nm width and, further on, even 10 nm width. Numerical difficulties that arose in the analytical calculation of the Cartesian oval when the change in refractive index across the lens surface is small, as is usual for X-ray optics, were overcome by a cubic approximation that was numerically stable. The focusing performance of lens surfaces following the cubic ‘X-ray’ approximation was compared with that of lens surfaces shaped either as ellipses or hyperbolas, or as parabolas, as previous authors have suggested. Elliptical or hyperbolic lens surfaces yield stronger peaks and lower tails at the focus than do parabolic lens surfaces, but surfaces that follow the cubic X-ray approximation provide better focal profiles than either. Examples taken from a proposed adiabatically focusing lens, in which the radius of curvature and the aperture of the lens surfaces both decrease along the beam direction, indicate that the advantages of the X-ray approximation over conic sections are most apparent in the final, most strongly curved, lenses.

## Figures and Tables

**Figure 8:**
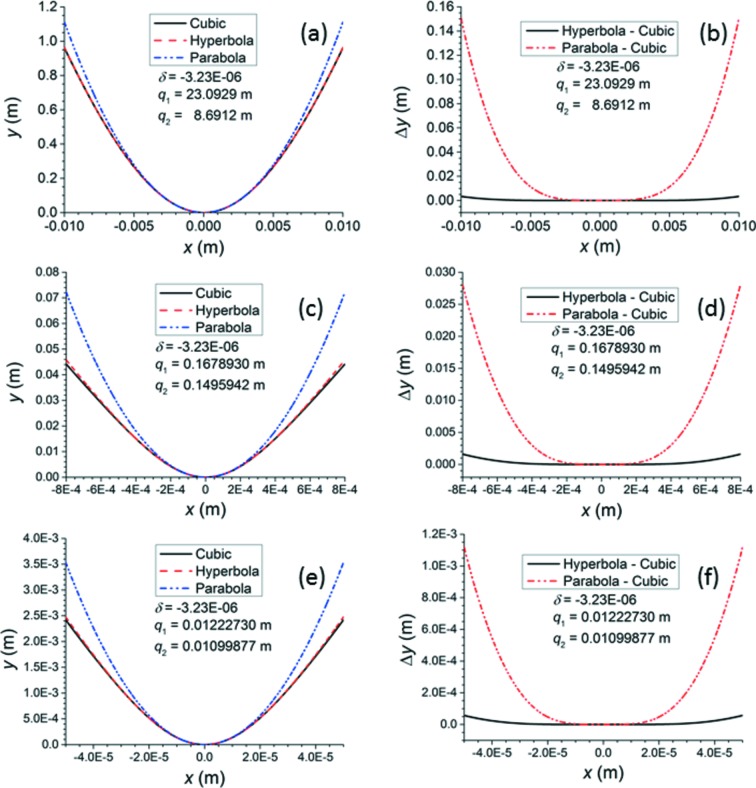
In all plots, 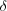 = 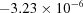. The label ‘Cubic’ means that the X-ray approximation of the ideal Cartesian oval was used to calculate the curve. Refer to Table 1 ▸ for the list of surfaces. (*a*, *c*, *e*) Comparison of cubic curve to paraxial hyperbola and parabola of surfaces 2, 24 and 48, respectively. (*b*, *d*, *f*) Deviation of paraxial hyperbola and parabola from cubic curve of surfaces 2, 24 and 48, respectively.

**Table 1:** Lens surfaces proposed for a diamond nanofocusing CRL for X-rays of energy 15 keV The distance from the apex of the last lens surface to the final focal plane is 11.017 mm. *i* is the place of each surface in the CRL. *R*_*i*_ is the radius at the apex. *f*_*i*_ is the focal length. *A*_*i*_ is the geometrical aperture. *q*_1*i*_ is the downstream distance of the object of lens surface *i*. (A negative value of *q*_1*i*_ means that the object of lens surface *i* is upstream.) *q*_2*i*_ is the downstream distance of the image of lens surface *i*. The distance between the apices of consecutive lens surfaces is 0.005 mm. For legibility, *R*_*i*_ and *A*_*i*_ are rounded to four significant digits, and *f*_*i*_, *q*_1*i*_ and *q*_2*i*_ are rounded to three decimal places.

*i*	*R*_*i*_ (mm)	*f*_*i*_ (mm)	*A*_*i*_ (mm)	*q*_1*i*_ (mm)	*q*_2*i*_ (mm)
1	0.05	15484.670	0.075	−46999.864	23092.905
2	0.045	13936.203	0.07425	23092.900	8691.196
3	0.0405	12542.583	0.07351	8691.191	5133.801
4	0.03645	11288.325	0.07277	5133.796	3528.896
5	0.03281	10159.492	0.07204	3528.891	2619.136
6	0.02952	9143.543	0.07132	2619.131	2035.944
7	0.02657	8229.189	0.07061	2035.939	1632.140
8	0.02391	7406.270	0.06990	1632.135	1337.408
9	0.02152	6665.643	0.06921	1337.403	1113.907
10	0.01937	5999.078	0.06851	1113.902	939.463
11	0.01743	5399.171	0.06783	939.458	800.220
12	0.01569	4859.254	0.06715	800.215	687.069
13	0.01412	4373.328	0.06648	687.064	593.780
14	0.01271	3935.995	0.06581	593.775	515.941
15	0.01144	3542.396	0.06516	515.936	450.345
16	0.01029	3188.156	0.06450	450.340	394.601
17	0.009265	2869.341	0.06386	394.596	346.891
18	0.008339	2582.407	0.06322	346.886	305.808
19	0.007505	2324.166	0.06259	305.803	270.245
20	0.006754	2091.749	0.06196	270.240	239.322
21	0.006079	1882.574	0.06134	239.317	212.325
22	0.005471	1694.317	0.06073	212.320	188.677
23	0.004924	1524.885	0.06012	188.672	167.898
24	0.004432	1372.397	0.05952	167.893	149.592
25	0.003988	1235.157	0.05893	149.587	133.428
26	0.003590	1111.641	0.05834	133.423	119.125
27	0.003231	1000.477	0.05775	119.120	106.446
28	0.002908	900.429	0.05718	106.441	95.189
29	0.002617	810.387	0.05660	95.184	85.179
30	0.002355	729.348	0.05604	85.174	76.268
31	0.002120	656.413	0.05548	76.263	68.325
32	0.001908	590.772	0.05492	68.320	61.238
33	0.001717	531.695	0.05437	61.233	54.909
34	0.001545	478.525	0.05383	54.904	49.253
35	0.001391	430.673	0.05329	49.248	44.194
36	0.001252	387.605	0.05276	44.189	39.667
37	0.001126	348.845	0.05223	39.662	35.613
38	0.001014	313.960	0.05171	35.608	31.981
39	0.0009124	282.564	0.05119	31.976	28.725
40	0.0008212	254.308	0.05068	28.720	25.806
41	0.0007390	228.877	0.05017	25.801	23.187
42	0.0006651	205.989	0.04967	23.182	20.837
43	0.0005986	185.390	0.04917	20.832	18.728
44	0.0005388	166.851	0.04868	18.723	16.834
45	0.0004849	150.166	0.04820	16.829	15.133
46	0.0004364	135.150	0.04771	15.128	13.605
47	0.0003928	121.635	0.04724	13.600	12.232
48	0.0003535	109.471	0.04676	12.227	10.999
